# Vicarious Exposure to Police Discrimination and Brutality, Concern, and Changes in Cigarette Smoking

**DOI:** 10.1007/s40615-025-02649-6

**Published:** 2025-12-03

**Authors:** Kiana Hacker, Kristen R. Hamilton-Moseley, Allana T. Forde, Julia Chen-Sankey, Kelvin Choi

**Affiliations:** 1 Division of Intramural Research, National Institute on Minority Health and Health Disparities, Bethesda, MD USA; 2 Center for Tobacco Studies, Rutgers Biomedical and Health Sciences, New Brunswick, NJ USA

**Keywords:** Vicarious exposure, Police discrimination, Police brutality, Concern, Cigarette smoking, Race and/or ethnicity

## Abstract

**Introduction:**

Police discrimination and brutality (PDB) disproportionately impacts racial and/or ethnic minority groups. However, it remains unclear how vicarious exposure to PDB varies in the USA and how it relates to concern and cigarette smoking across racial and/or ethnic groups.

**Methods:**

Data were from a survey conducted in January–February 2021 with a US nationally representative sample of adults (≥ 21 years) who reported current and recent former (within the past 12 months) commercial tobacco use (*n* = 1700). Associations among self-reported vicarious exposure to PDB, concerns about PDB, and changes in cigarette smoking behaviors were examined.

**Results:**

Black/African American individuals were more likely than White individuals to report frequent vicarious exposure to PDB (adjusted odds ratio (aOR) = 1.58), 95% confidence intervals (95% CI) = 1.01, 2.46). Average concern about PDB depended both on race and/or ethnicity and frequency of vicarious exposure to PDB. Participants reporting frequent vicarious exposure to PDB had higher concern compared to participants reporting nonfrequent vicarious exposure, with the largest differences observed in Black/African American and Asian participants (interaction *p*-value < 0.05). Overall, concern associated with changes in cigarette smoking: each increment of concern associated with greater odds of progressing to past-30-day smoking (aOR = 1.55, 95% CI = 1.15–2.10) and increased monthly cigarette consumption (adjusted regression coefficient (aRC) = 13.58 cigarettes/month, 95% CI = 1.02–26.14). These associations varied somewhat by race and/or ethnicity.

**Conclusions:**

Vicarious exposure to PDB was prevalent, especially among Black/African American individuals. These exposures may have heightened concerns about PDB in the entire population and may in turn have worsened cigarette-smoking behaviors, with some variation by race and/or ethnicity.

## Introduction

Police brutality refers to the use of physical, psychological, or other forms of force or coercion that go beyond the needs and expectations of the job [1]. Between 1980 and 2019, an estimated 30,800 deaths resulted from police brutality in the USA, with the age-standardized mortality rate increasing from 0.25 per 100,000 in the 1980 s to 0.34 per 100,000 in the 2010 s [2]. During that time period, the mortality rate related to police brutality was higher among Black people (0.69 per 100,000) and Hispanic people (0.35 per 100,000) than among White people (0.20 per 100,000) [2].

More than half of US adults in 2021 said that police are more likely to use deadly force against Black people than White people [3], coincided with mortality data, as over the course of the lifetime, Black men are 2.5 times more likely than White men, and Black women are 1.4 times more likely than White women, to be fatally shot by the police [4, 5]. Hispanic and Native American individuals also are more likely to be fatally shot by police compared to White individuals [5–7]. Absent deadly force, police discrimination and brutality (PDB) can take different forms, including harassment, violence, using racial slurs, threatening use of force, pointing a gun, and raiding homes, as well as neglectful policing (e.g., failure to respond when called) [8]. In 2020, the American Medical Association deemed police brutality a public health concern and called on researchers to study the public health effects of police brutality [9].

The detrimental health effects of PDB can go beyond those who experience it directly to also impact those who are exposed to it indirectly. Vicarious (i.e., indirect) exposure to PDB can occur in many ways (e.g., in-person witnessing, social media, community exposures, personal communication with friends and family members) and is associated with psychological harms [10]. Adolescents who reported directly or vicariously experiencing police stops had worse self-reported health compared to those who reported having no contact with police, especially among Black and Hispanic individuals [11], suggesting that vicarious exposure to PDB can have negative health consequences. Lab-based experiments also indicated that vicarious exposure to PDB was associated with greater cardiac sympathetic reactivity and negative affect among Black young adults [12] and activated stress responses to a larger extent in Black than White Americans [13] Furthermore, each additional killing of an unarmed Black American by police in their state was associated with an increase in the number of days reported having poor mental health in the previous month among Black American adults [10].

There is a growing literature on mental health symptomatology and anticipatory stress of police brutality in adults and adolescents [14–16]. In work by Alang and colleagues [14, 16], anticipatory stress of police brutality was associated with greater odds of anxiety and depression in adults, and the association of depressed mood with anticipatory stress was stronger in Black and Latino respondents compared to White respondents. Adolescents with anticipatory stress of police brutality had more symptoms of depression, anxiety, and PTSD than adolescents without such stress [15]. Taken together, these studies suggest that vicarious exposure to PDB and anticipatory stress of PDB can have large health impacts on racial and/or ethnic minority groups.

Vicarious exposure to PDB also might be associated with increased substance use. Among Black and Hispanic youth and young adults in Los Angeles County, distress about police brutality is associated with increased substance use [17–19]. Among adults who smoke cigarettes, psychological distress is associated with stress-induced cigarette smoking [20]. Racial and/or ethnic minority groups bear a disproportionate burden of smoking-related health conditions [21, 22]. For example, Black Americans have a high Disability-Adjusted Life Years (DALYs) rate due to smoking-related cancer compared to White Americans [23]. Compared with White adults, racial and/or ethnic minority adults were less successful in quitting smoking and had higher smoking-associated medical spending [24]. Consequently, it is important to understand how vicarious exposure to PDB may impact cigarette smoking by race and/or ethnicity.

Therefore, the present study examined associations of vicarious exposure to PDB with concerns about PDB and changes in cigarette smoking behaviors in a nationally representative sample of adults who reported currently or formerly using tobacco products. Black/African American and Hispanic/Latino individuals were expected to be more likely than White individuals to report frequent vicarious exposure to PDB. They were also expected to report higher levels of concern about PDB compared to their White counterparts. In addition, we expected concerns about PDB to be associated with progression of cigarettes, continuation of smoking, and increased cigarette consumption. We further anticipated that these associations would be stronger among Black/African American and Hispanic/Latino individuals than White individuals.

## Methods

### Study Population

Data were from a survey conducted by YouGov, Inc., from January to February 2021, containing a US nationally representative sample of adults (≥ 21 years) who reported current and recent former (within the past 12 months) commercial tobacco use (*n* = 1700). YouGov is an online survey panel that uses a sample matching approach to achieve national representation. YouGov used national survey data (e.g., American Community Survey) to create a sampling frame that met the inclusion criteria of this study and then used the sampling frame to recruit a matching sample from its panelists via email invitations, oversampling Black/African American and Asian individuals. This sample matching approach can achieve, after statistical weighting, the same level of representation as the random-digit-dialing sampling approach [25]. Panelists screened to be eligible for this study were invited to complete the survey after providing informed consent. They were compensated according to YouGov policy. Of the 2404 panelists who were eligible for this study, 2123 completed the survey (cooperation rate was 88.3% among those who were screened eligible). Surveys with inconsistent responses (e.g., surveys endorsing never using a tobacco product after having endorsed using the product in a previous item) were excluded to meet the predetermined study sample size. Because this analysis involved de-identified data, it was considered not human subjects research and did not require review or approval by the National Institutes of Health Institutional Review Board.

### Measures

#### Vicarious Exposure to Police Discrimination and Brutality (PDB)

To assess the frequency of vicarious exposure to PDB, participants were asked “How often do you hear stories about someone being discriminated against, harassed, treated unfairly, or killed by the police?”. There were six response options ranging from “almost every day” to “never” that were later dichotomized into frequent and infrequent vicarious exposure to police brutality. *Frequent vicarious exposure to PDB* included the response options “Almost every day (weighted % = 15.8%),” “At least once a week (weighted % = 17.8%),” and “A few times a month (weighted % = 25.9%)”, and *infrequent vicarious exposure to PDB* included the response options “A few times a year (weighted % = 21.4%),” “Less than once a year (weighted % = 5.9%),” and “Never (weighted % = 12.3%).”

#### Concerns About PDB

A published measure of concerns about discrimination [26] was adapted to assess participants’ concerns about police discrimination and brutality. Participants were asked about their levels of concern, worry, and stress regarding PDB in three separate items: “Are you concerned/worried/stressed that you or your family may be discriminated against, harassed, treated unfairly, or killed by the police, just like in these stories?” (Refer to stories about someone being discriminated against, harassed, treated unfairly, or killed by the police.) Response options for each item (i.e., concern, worry, stress) were assigned numerical values: 1 = not at all, 2 = slightly, 3 = somewhat, 4 = very, and 5 = extremely. Responses were averaged (ratings 1–5) across these three items to represent participants’ overall level of concern about police brutality. These three items had very high internal consistency (Cronbach alpha = 0.95).

#### Cigarette Smoking Behaviors

To assess cigarette smoking behaviors, participants were asked about their history of smoking cigarettes. Responses included “Never,” “Used before, but not now,” “Currently using, some days,” and “Currently using, every day.” Participants who reported currently smoking cigarettes some days or every day were asked to answer a follow-up question: “In the past 30 days, on how many days did you smoke cigarettes?” Response options ranged from 0 to 30 days. Participants who reported smoking at least one day in the past 30 days were categorized as engaging in past-30-day smoking at the time of the survey; all others were categorized as not engaging in past-30-day smoking. Of those who reported past-30-day smoking, participants reported the average number of cigarettes smoked on the days in which they smoked. Monthly cigarette consumption at the time of the survey was calculated by multiplying the number of days smoked in the past 30 days and the number of cigarettes per day.

Participants also reported their experiences with smoking cigarettes 12 months prior to the survey, and their responses included “Never,” “Used before, but not during that time,” “Used some days,” and “Used every day.” Those who reported smoking cigarettes some days or every day 12 months prior to the survey further reported the number of days they smoked cigarettes during a 30-day period at that time and the number of cigarettes they smoked per day on the days they smoked. Using the same approach as in categorizing past-30-day smoking and monthly cigarette consumption at the time of survey, we categorized participants into “past-30-day smoking” or “not smoking 12 months prior to the survey” and calculated monthly cigarette consumption 12 months prior to the survey in number of cigarettes per month.

Information on cigarette smoking at the time and 12 months prior to the survey was used to derive changes in cigarette smoking behaviors. Among those who reported no past-30-day smoking 12 months prior to the survey, the behavioral outcome was *Progression to past-30-day cigarette smoking,* defined as reporting past-30-day cigarette smoking at the time of the survey (vs. continuing to report no past-30-day smoking). Among those who reported past-30-day cigarette smoking 12 months prior to the survey, the behavioral outcome was *Continuation of past-30-day cigarette smoking*, defined as also reporting past-30-day smoking at the time of the survey (vs. continuing to report no past-30-day cigarette smoking at the time of survey). Lastly, among those who reported past-30-day cigarette smoking both 12 months prior and at the time of the survey, *changes in monthly cigarette consumption* were calculated by subtracting the monthly cigarette consumption 12 months prior to the survey from monthly cigarette consumption at the time of the survey.

*Covariates* included age, sex, race and/or ethnicity, highest level of education, annual household income, and marital status. Race and/or ethnicity was self-reported by participants using predetermined categories which were categorized into Hispanic/Latino, Asian, Black/African American, White, and Other (i.e., American Indian, Alaska Native, Middle Eastern or North African, Pacific Islander, multiracial/multiethnic, and other) because of small sample sizes in these groups. Participants also reported whether they were ever treated unfairly by police since March 2020 (a time characterized by protests and increased media reports on police brutality following George Floyd’s death). These covariates were included because of their relationship with vicarious exposure to PDB and/or cigarette smoking.

### Statistical Analyses

Data were weighted to be nationally representative within racial and/or ethnic groups. First, the frequencies and percentages of select population characteristics (e.g., demographic information) in the total population were calculated. Second, the prevalence of frequent vicarious exposure to PDB by population characteristics was estimated. Third, multivariable logistic regression was conducted to assess whether vicarious exposure to PDB varied across racial and/or ethnic groups, adjusting for demographics and whether they were ever treated unfairly by police. Fourth, mean level of concern about PDB was calculated in the overall study population and across racial and/or ethnic groups. Fifth, multivariable linear regression was used to compare concerns about PDB across racial and/or ethnic groups, adjusting for covariates. Sixth, multivariable logistic regression was used to test the associations between concern about PDB with progression to past-30-day smoking, continuation of past-30-day smoking, and changes in monthly cigarette consumption, adjusting for covariates, in the overall study population and across racial and/or ethnic groups. Analyses were conducted in 2024 using SAS® Enterprise version 9.4 (SAS Institute: Cary, NC).

## Results

The weighted demographic characteristics of US adults who reported current and recently former commercial tobacco use in 2021 are presented in Table 1. Overall, 59.7% of these individuals reported frequent vicarious exposure to PDB. Exposure varied significantly by race and/or ethnicity, with Black/African American individuals most likely to report frequent exposure to PDB, followed by Hispanic/Latino individuals, Asian individuals, White individuals, and individuals of other races (Table 1). Black/African American individuals (compared to White individuals) and those who were ever treated unfairly by police (compared to never) had higher odds of frequent vicarious exposure to PDB (*p* < 0.05, Table 1).

**Table 1:** Weighted demographic characteristics, prevalence of frequent vicarious exposure to police discrimination and brutality, and their associations

Population characteristics	Overall weighted %	Frequent vicarious exposure to police discrimination and brutality	
		Weighted %	Adjusted odds ratio (95% confidence interval)
**Race and/or ethnicity**			
Asian	4.5	59.1	0.89 (0.50, 1.61)
Black/African American	13.6	69.7	**1.58 (1.01, 2.46)**
Hispanic/Latino	13.1	62.5	1.09 (0.70, 1.68)
Other	2.4	44.3	0.54 (0.23, 1.26)
White	66.4	57.6	Reference
**Sex**			
Female	40.9	63.0	1.34 (0.97, 1.84)
Male	59.1	57.3	Reference
**Age (years)**			
21–30	27.2	67.1	1.54 (0.95, 2.42)
31–45	32.5	61.7	1.18 (0.81, 1.73)
> 45	40.3	53.4	Reference
**Annual household income**			
< $50,000	60.6	61.3	1.11 (0.77, 1.60)
≥ $50,000	39.4	57.6	Reference
**Education**			
≤ High school/GED	47.8	55.2	0.66 (0.42, 1.06)
Some college	33.6	65.6	1.16 (0.75, 1.80)
≥ Bachelor’s degree	18.6	60.3	Reference
**Marital status**			
Married	37.0	56.0	Reference
Domestic/civil partnership	10.9	66.8	1.38 (0.75, 2.53)
Never married	33.5	61.1	0.96 (0.64, 1.44)
Separated, divorced, widowed	18.6	60.1	1.27 (0.81, 2.01)
**Treated unfairly by police**			
Yes	31.7	71.6	**2.01 (1.37, 2.95)**
No	68.3	54.1	Reference

*AOR* adjusted odds ratio, *CI* confidence interval. Estimates are adjusted for all variables in the table. Bolded estimates are statistically significant (*p *< 0.05)

Overall, the mean level of concern about PDB was 2.10 (95% CI = 2.04–2.16). This mean level varied by race and/or ethnicity: Black/African American individuals had a mean level of 2.83 (95% CI = 2.60–3.07), followed by Asian individuals (2.39, 95% CI = 2.10–2.67), Hispanic/Latino individuals (2.34, 95% CI = 2.17–2.52), individuals of other races/ethnicities (1.93, 95% CI = 1.66–2.21), and White individuals (1.61, 95% CI = 1.51–1.70). Asian, Hispanic/Latino, and Black/African American individuals (compared to White individuals), younger adults aged 21 to 45 (compared to > 45 years and older), those ever treated unfairly by police (compared to never), and those who reported frequent vicarious exposure to PDB (compared to infrequent) had higher levels of concern about police discrimination and brutality (Table 2).

**Table 2:** Associations of demographics, ever treated unfairly by police, and frequent vicarious experience of police discrimination and brutality with levels of concern for police discrimination and brutality

Variable	Adjusted regression coefficient	95% confidence interval	
**Race and/or ethnicity**			
Asian	**0.58**	**0.33**	**0.83**
Black/African American	**0.95**	**0.73**	**1.16**
Hispanic/Latino	**0.50**	**0.29**	**0.71**
Other	0.24	−0.04	0.54
White	Reference		
**Sex**			
Female	−0.01	−0.15	0.12
Male	Reference		
**Age (years)**			
21–30	0.14	−0.05	0.33
31–45	**0.22**	**0.06**	**0.38**
> 45	Reference		
**Annual household income**			
< $50,0000	0.06	−0.09	0.21
≥ $50,000	Reference		
**Education**			
≤ High school/GED	−0.02	−0.19	0.16
Some college	0.04	−0.13	0.21
≥ Bachelor’s degree	Reference		
**Marital status**			
Married	0.08	−0.21	0.38
Domestic/civil partnership	0.04	−0.12	0.20
Never married	−0.15	−0.34	0.03
Separated, divorced, widowed	Reference		
**Treated unfairly by police**			
Yes	**0.91**	**0.72**	**1.10**
No	Reference		
**Frequent vicarious experience of police brutality**			
Yes	**0.39**	**0.26**	**0.52**
No	Reference		

Bolded estimates are statistically significant (*p *< 0.05)

**Fig. 1:**
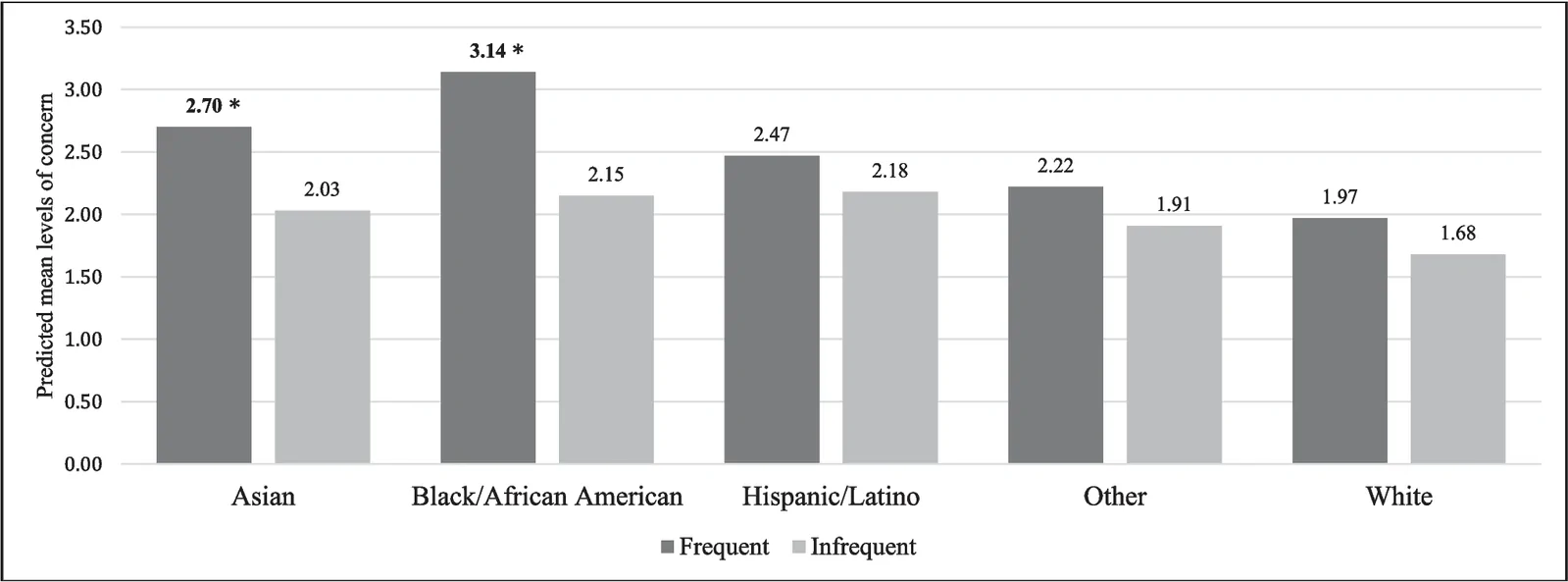
Predicted mean levels of concern for police brutality by frequent vicarious experience of police discrimination and brutality and race and/or ethnicity. Adjusted for age, sex, annual household income, education, marital status, and treated unfairly by police. Bolded estimates with asterisks are statistically different between frequent and infrequent vicarious exposure of police discrimination and brutality within the racial and/or ethnic group

Mean concern about PDB depended both on race and/or ethnicity and frequent vicarious exposure to PDB (interaction F-test = 3.33, df = 4, *p* = 0.01). Specifically, frequent vicarious exposure to PDB was associated with a 0.99 increase in concern about police discrimination and brutality among Black/African American individuals (*t* value = 5.274, *p* < 0.01), a 0.67 increase among Asian individuals (*t* value = 2.84, *p* < 0.01), a 0.29 increase among White individuals (*t* value = 3.68, *p* < 0.01), and no statistically significant increase among Hispanic/Latino individuals and individuals of other races (*t* value = 1.05, *p* = 0.29, Fig. 1).

Among participants who reported no past-30-day cigarette smoking 12 months prior to the survey, 18.2% progressed to past-30-day cigarette smoking at the time of the survey, with significant variation by race and/or ethnicity (Table 3). In US adults who reported current and recently former commercial tobacco use in 2021, every point increase in concern about PDB was associated with a 55% increase in the odds of progression to past-30-day smoking. The race and/or ethnicity-specific analyses indicated that this association was statistically significant for Hispanic/Latino, Asian, and White individuals, although confidence intervals were wide for Asian individuals. Among those who reported past-30-day cigarette smoking 12 months prior to the survey, 84.0% continued past-30-day smoking at the time of the survey; this association also varied by race and/or ethnicity. Despite the lack of a statistically significant association overall, every point increase in concern about PDB was associated with a 120% increase in the odds of continuing past-30-day smoking among Black/African American individuals but a 50% decrease in the odds among Asian individuals. It is noteworthy that only 21 Asian individuals discontinued past-30-day smoking, making the estimated association less reliable. Lastly, among those who reported past-30-day smoking both 12 months prior and at the time of the survey, the average monthly cigarette consumption reduced by 23.35 cigarettes (median change = 0 cigarettes). As expected, in the overall sample, every point increase in concern about PDB was associated with increased monthly cigarette consumption by 13.58 cigarettes. In race and/or ethnicity stratified analysis, this association was observed among White individuals, while individuals belonging to other races showed a decrease in monthly cigarette consumption associated with higher concern about PDB. The reliability of the latter finding was questionable since it was based on only 31 individuals.

**Table 3:** Associations between concerns for police discrimination and brutality and changes in cigarette smoking behaviors

	Reported no past-30-day use at 12 months prior to the survey		Reported past-30-day use at 12 months prior to the survey		Reported past-30-day use at 12 months prior and time of survey
Race and/or ethnicity	Outcome: progression to past-30-day smoking	Adjusted odds ratio of concern about police discrimination and brutality with the outcome (95% confidence interval)	Outcome: continuation of past-30-day smoking	Adjusted odds ratio of concern about police discrimination and brutality with the outcome (95% confidence interval)	Adjust regression coefficient of concern about police discrimination and brutality with changes in monthly cigarette consumption among those continued smoking (95% confidence interval)
Cigarette smoking					
Overall	18.2%	**1.55 (1.15, 2.10)**	84.0%	1.11 (0.83, 1.47)	**13.58 (1.02, 26.14)**
Hispanic	31.1%	**2.11 (1.21, 3.69)**	76.9%	1.21 (0.52, 2.83)	25.03 (−14.34, 64.39)
Asian	9.6%	1.73 (0.82, 3.70)	71.0%	**0.50 (0.30, 0.83)**	−7.75 (−45.96, 30.47)
Black	36.0%	0.77 (0.40, 1.48)	88.5%	**2.20 (1.09, 4.78)**	9.52 (−12.17, 31.21)
Other	6.3%	Non−estimable	81.1%	Non−estimable	**−91.53 (−166.36, −16.70)**
White	12.6%	**2.14 (1.27, 3.58)**	85.1%	1.01 (0.69, 1.48)	**19.73 (3.54, 35.92)**

Note: All estimates were adjusted for age, sex, race and/or ethnicity, highest level of education, annual household income, marital status, ever treated unfairly by police. Bolded estimates are statistically significant (*p* < 0.05)

## Discussion

This study examined vicarious exposure to police discrimination and brutality (PDB) in a nationally representative sample of US adults in early 2021. Black/African American individuals were more likely than White individuals to report frequent vicarious exposure to PDB. Given that Black/African American young men face the highest risk of being killed by the police compared to all other racial and/or ethnic groups [5], Black individuals may be more likely than White individuals to pay attention to and recall these reports. Accounts of PDB incidents by friends and family members may be more salient to Black individuals. Additionally, previous work has reported elevated rates of direct exposure to PDB in other racial and/or ethnic individuals compared to White individuals [5, 27], and many other racial and/or ethnic groups are vicariously exposed to PDB through multiple sources including personal communication with friends and family members, social media, and news reports [28]. This may, in part, explain why higher levels of concern about police discrimination and brutality were observed among Asian, Black/African American, and Hispanic/Latino individuals compared to White individuals [29].

Across all racial and/or ethnic groups, concern about PDB was greater in those who reported frequent vicarious exposure to PDB (compared to those who reported infrequent vicarious exposure), especially among Asian, Black/African American, and White individuals. Based on these findings, vicarious exposure to PDB may affect *all* racial and/or ethnic groups in the USA, including White individuals, who are the least likely racial and/or ethnic group to directly experience these incidents [5]. Therefore, reducing and eliminating police discrimination and brutality could benefit *all* racial and/or ethnic groups in the USA.

It is noteworthy that the strongest associations between frequent exposure to PDB with concern about PDB were observed in Black/African American and Asian individuals. These findings suggest that given the same frequent vicarious exposure, certain racial and/or ethnic groups may bear higher levels of psychological distress than other groups. Since Black/African American people are more likely to experience police violence [5], Black/African American individuals are likely to see or hear about Black/African American victims in vicarious exposure to PDB, and such events may have added salience for Black/African American individuals, resulting in heightened distress. Similarly, while Asian individuals as a whole experience less frequent police brutality than Black/African American individuals, research has found that some Asian subgroups have elevated risk of fatal police violence [30]. Awareness of these differences may contribute to heightened concern about PDB in Asian individuals.

As expected, concern about PDB was associated with *greater* odds of progression to past-30-day smoking (which could represent intensifying smoking frequency or returning to cigarette smoking among those who had quit) overall and in Hispanic/Latino and White individuals, *greater* odds of continuation of past-30-day smoking among Asian and Black/African American individuals, and increased monthly cigarette consumption in both White individuals and adults who use tobacco products in general. It is possible that individuals smoke cigarettes as a maladaptive behavioral response to psychological stress and anxiety associated with having concerns about PDB. However, this may not fully explain the variation by race and/or ethnicity [31]. Previous studies also showed that tobacco retailer density was higher in neighborhoods with a higher proportion of Black and Hispanic residents [32], while Black and Hispanic adults who smoke cigarettes were less likely than their White counterparts to use cessation treatments when quitting smoking [33]. As vicarious exposure to PDB could induce stress and potentially lead to stress-induced smoking, availability of cigarettes in the neighborhood and access to effective smoking cessation treatment may play a role in the racial and/or ethnic difference in the relationships between concerns for PDB and smoking behaviors in this study. The negative association between concerns for PDB and changes in monthly cigarette consumption among those who were categorized as “other” race was unexpected. This was likely because of the small sample size of this group, reflected in the large confidence interval surrounding the adjusted regression coefficient. Future studies are needed to confirm this finding.

A two-pronged approach could be used to address PDB. First, reduce police misconduct and over-policing. Steps to reduce police misconduct may include requiring officers to receive relevant training [34] and reducing the over-policing of neighborhoods that are predominantly comprised of racial and/or ethnic minority residents [35]. This could reduce both the direct and vicarious exposure to PDB. Second, healthcare providers could screen for and address frequent vicarious experiences of PDB. For example, items used in this study can be integrated as part of the patient intake form so that healthcare providers can follow up with those with high levels of concern for PDB. Moreover, tobacco cessation specialists could screen for PDB exposure and concern during an initial assessment and incorporate discussion of PDB exposure as triggers for cigarette smoking and, if needed, refer these individuals for further counseling.

### Limitations

First, the study population only included individuals who currently or formerly used commercial tobacco products. Generalizability of our findings to individuals who never used commercial tobacco is unclear. Second, we were unable to further disaggregate racial and/or ethnic groups (e.g., differentiating African American, Afro-Caribbean, or African in the Black/African American racial and/or ethnic group). Third, the survey was conducted in English only, which precludes non-English speaking individuals from participating. Fourth, this survey was completed online, requiring participants to access the Internet through a computer or phone, which could preclude individuals without internet access from participating in the study who may have high vicarious exposure to PDB and cigarette smoking (e.g., inmates). Fifth, the current study only measured vicarious police exposure as hearing stories about PDB, and other sources of vicarious exposures were not explicitly measured, a limitation that could lead to under-reporting of vicarious discrimination exposures. Future work could include a more detailed assessment of exposures to vicarious discrimination, including from multiple sources. Sixth, given the cross-sectional design of this study, we cannot infer causality. Seventh, despite oversampling, the number of Asian participants in the study was still limited. This results in limited statistical power for some of the statistical tests, especially interaction tests, reflected in wide confidence intervals in some of our estimates. Eighth, the study did not measure lifetime exposure to unfair treatment by the police, which could be an unmeasured confounder. Ninth, dichotomization of PDB exposure may have oversimplified the complex experience of PDB exposure, both directly and vicariously. Finally, since PDB exposure and concern, as well as smoking behaviors were measured through self-report, there were likely recall errors.

## Conclusions

This study found that vicarious exposure to PDB was prevalent, especially among Black/African American individuals. These experiences were related to heightened concern about PDB in the entire population of interest. Additionally, concern about PDB was associated with worsening of cigarette smoking behaviors. If future longitudinal studies confirm our findings, addressing PDB could improve well-being of all races and ethnicities, especially among racial and/or ethnic minority populations.

## Data Availability

Data are available for research purposes per request. Please contact the corresponding author for data access request and approval.
